# Time of onset of pre-eclampsia as a determinant of risk of cardiovascular disease and renal impairment at six weeks post partum: a cohort study in Lagos, Nigeria

**DOI:** 10.1136/bmjopen-2024-094397

**Published:** 2025-07-15

**Authors:** Olaniyi Araotan Kusamotu, Ochuwa Adiketu Babah, Ifeoma Udenze, Ayodeji A Oluwole, Bosede Bukola Afolabi

**Affiliations:** 1 Department of Obstetrics and Gynaecology, Lagos University Teaching Hospital, Surulere, Nigeria; 2 Department of Obstetrics and Gynaecology, University of Lagos College of Medicine, Lagos, Nigeria; 3 Department of Global Public Health, Karolinska Institute, Solna, Sweden; 4 Centre for Clinical Trials, Research and Implementation Science, University of Lagos College of Medicine, Idi-Araba, Lagos, Nigeria; 5 Department of Chemical Pathology, University of Lagos, Akoka, Nigeria

**Keywords:** Cardiovascular Disease, Blood Pressure, Hypertension, Maternal medicine

## Abstract

**Abstract:**

**Objectives:**

Pre-eclampsia causes significant maternal and perinatal morbidity and mortality. It also causes changes in the cardiovascular, endothelial and metabolic systems, from which women may not fully recover after delivery. This study examined the association between the time of onset of pre-eclampsia and the risk for cardiovascular disease (using glucose tolerance, lipid profile and blood pressure) and renal function at 6 weeks post partum.

**Study design:**

A prospective cohort study.

**Setting:**

Lagos University Teaching Hospital, Idi-Araba, Mother and Child Centre, Gbaja, Surulere and Lagos Island Maternity Hospital, Lagos, Nigeria.

**Participants:**

44 women with pre-eclampsia were studied and data on their sociodemographic characteristics, gestational age at diagnosis and blood pressure were collected on admission. They were followed up through delivery till 6 weeks post partum, when blood pressure check, 75 g oral glucose tolerance test, fasting lipid profile and serum creatinine were done.

**Outcome measures:**

The exposure was pre-eclampsia. The outcomes were cardiovascular disease risk markers, viz persistent hypertension, glucose intolerance and dyslipidaemia, and renal function at 6 weeks post partum in women who had pre-eclampsia.

**Statistical analysis:**

Data were analysed using Stata V.16.1. Mann Whitney-U test was used to compare medians and Fisher's exact test was used to compare the categorical variables.

**Results:**

Of the women studied, 13 (29.5%) had early onset pre-eclampsia and 31 (70.5%) had late onset pre-eclampsia. Mean gestational age at diagnosis was 30.8±1.57 weeks in women with early-onset pre-eclampsia and 35.6±1.26 weeks in women with late-onset pre-eclampsia (p<0.001). There was no significant difference in terms of disease severity in women who had early-onset compared with late-onset disease. Of the cardiovascular risk markers assessed at 6 weeks post partum, only serum triglyceride was found to be statistically higher in women with early onset compared with late onset pre-eclampsia, median values of 135 mg/dL (130-182 mg/dL) vs 128 mg/dL (121-139 mg/dL), p = 0.008. Only eight (18.2%) of the pre-eclamptic women became normotensive at the end of puerperium.

**Conclusion:**

The prevalence of persistent hypertension at 6 weeks post partum is high in women with pre-eclampsia. Serum triglyceride concentration was significantly higher in early onset compared with late onset pre-eclampsia; subsequent studies powered to determine the full cardiovascular risk and how long to follow postnatal women up will be beneficial.

**STRENGTHS AND LIMITATIONS OF THIS STUDY**

- This was a prospective cohort study which afforded us an opportunity to have a more complete data collection; more so, none of the participants in this study were lost to follow-up likely because they were regularly reminded of their appointments.
- There was a paucity of studies on a similar topic, especially in sub-Saharan Africa at the time this study was commenced, so data from an earlier study conducted in Utrecht, The Netherlands were thus relied on for sample size calculation. This accounted for the low statistical power in this study. In addition, the small number of participants in the early onset pre-eclampsia group limits the interpretation of the findings.
- This study adopted a consecutive sampling technique which is non-probabilistic.
- Apart from blood pressure that was measured at enrolment and at 6 weeks post partum, all the biomarkers for cardiovascular and renal disease risk assessments were measured only at the 6 weeks postpartum time point. It would have been good to have the biomarkers measured at baseline because some women might have asymptomatic or underdiagnosed medical conditions related to these biomarkers.

## Introduction

 Pre-eclampsia is a multisystemic disorder of pregnancy characterised by new onset hypertension occurring after 20 weeks gestation in pregnant women who were previously non-hypertensive and had at least one associated complication like proteinuria, organ dysfunction or impaired uteroplacental circulation.^1^ The worldwide prevalence is estimated to be 2–10% of all pregnancies.^2^,^4^ In Nigeria, it occurs in 2–16% of pregnancies.^2^,^4^ It is well known that the severity of pre-eclampsia correlates with maternal and perinatal morbidity and mortality.^5 6^ Currently, the indicators of disease severity include blood pressure (BP) levels, mean arterial blood pressure, degree of proteinuria, symptomatology and laboratory parameters like serum uric acid, platelet count, coagulation factor and elevated liver enzymes.^7^

Pre-eclampsia, being a systemic disorder, is known to be characterised by various manifestations of organ dysfunction, chief among them being hypertension and proteinuria, which are signs of renal dysfunction. Evidence has also shown that changes may occur in the cardiovascular, endothelial and metabolic systems during pre-eclampsia, from which patients may not fully recover after delivery and thus predispose to future cardiovascular disease.^8^ The metabolic risk factors for cardiovascular diseases may manifest in individuals as raised BP, impairments in blood glucose levels or deranged lipid profile, while the risk of renal dysfunction may manifest as deranged serum creatinine levels or persistent proteinuria. Fortunately, these risk factors are measurable.

We routinely discharge all patients from the postnatal clinic at the end of the puerperium. The puerperium generally lasts 6 weeks and is the period of adjustment after delivery when the anatomical and physiological changes of pregnancy are reversed, and the body returns to the normal, non-pregnant state.^9^ In sub-Saharan Africa, postnatal care is suboptimal,^10^ and it has also not been extensively investigated whether these differences in cardiovascular risk factors may be related to gestational age at onset of pre-eclampsia. A recent pilot study conducted in our centre found the incidence of persistent hypertension in women with pre-eclampsia to be 13.4%, at 6 weeks post partum.^11^ We therefore hypothesised that some other markers of cardiovascular or renal risk, such as deranged glucose or lipid profile, and serum creatinine may also still be abnormal at the same time. We also decided to examine whether the time of onset of pre-eclampsia has a differential effect on these markers, as this would enable further determination of the risks associated with early or late pre-eclampsia.

This study sought to determine the association between time of onset of pre-eclampsia (early vs late onset) and cardiovascular risk markers such as hypertension, dyslipidaemia and deranged blood glucose and renal function at the end of puerperium.

## Methodology

### Study design and setting

It was a prospective cohort study conducted at Lagos University Teaching Hospital, Mother and Child Centre, Gbaja, Surulere, Lagos and Lagos Island Maternity Hospital, Lagos between March 2021 and September 2021. These health facilities were purposively selected because of their high obstetric patient load and close proximity to one another to facilitate effective coordination of the research. They are tertiary and secondary levels of healthcare delivery, respectively, with consultant-led obstetric teams and are situated in the heart of Lagos Mainland. They serve Lagos state and its environs and are open to all categories of women. Their obstetric units comprise consultant-led teams with vast experience in the management of pre-eclampsia. Referrals are received from both private and public hospitals in Lagos and other parts of the country.

### Study population

The study population were all pregnant women with pre-eclampsia irrespective of the time of onset and who gave informed written consent. Excluded from this study were pregnant women with previously diagnosed renal or cardiovascular disease including chronic hypertension, endocrine diseases such as diabetes mellitus, thromboembolic disease and chronic liver disease and women with multiple gestation. The diagnosis of pre-eclampsia was made when a pregnant woman after 20 weeks gestation had a BP ≥140/90 mm Hg, measured on at least two occasions with an interval of at least 4 hours apart and significant proteinuria. Significant proteinuria was defined as greater than >0.3 g of urinary protein excretion per 24 hour urine collection or ≥2+ on dipstick on at least two occasions (≥4 hours apart). In our clinical experience, the majority of pregnant women with pre-eclampsia were often delivered before completion of urine collection in 24 hours, often due to the severity of disease. A dipstick assay was used in the diagnosis of pre-eclampsia.

### Definition of terminologies

For the purpose of this study, severe pre-eclampsia is defined as the presence of at least one of the following in women diagnosed with pre-eclampsia: a systolic BP ≥160 mm Hg, a diastolic BP≥110 mm Hg, proteinuria ≥5 g in 24 hours, proteinuria of 3+or more on dipstick test, oliguria (<500 mL/24 hours) or pulmonary oedema.^12^ Also, cases with haemolysis, elevated liver enzymes and low platelet count (HELLP) syndrome, elevated liver enzymes accompanied by right upper quadrant pain, persistent cerebral symptoms such as headache or blurry vision and platelet count <1 00 000/mL were considered severe. Uteroplacental dysfunction (such as fetal growth restriction, abnormal umbilical artery Doppler wave form analysis or stillbirth) in the presence of elevated BP was considered severe pre-eclampsia.^13^ Proteinuria of 2+ (in addition to elevated BP <160/110 mm Hg) was classified as mild pre-eclampsia and that with BP >160/100 mm Hg was classified as severe. Proteinuria ≥3+ with elevated BP >140/90 mm Hg was classified as severe pre-eclampsia. Urinary output <25 mL per hour was classified as severe pre-eclampsia. Participants with pulmonary oedema on chest examination were classified as having severe pre-eclampsia.

Gestational age was determined from the last menstrual period or from the earliest ultrasound scan done if the last menstrual period is unknown. Pre-eclampsia occurring before 34 weeks of gestation was regarded as early onset and that occurring at 34 weeks gestation and above was regarded as late onset pre-eclampsia.^5 13^

Dyslipidaemia was defined as the derangement of one or more of the lipoproteins in blood, such as elevated total cholesterol (TC), low density lipoprotein (LDL) and/or triglycerides (TGs) or low levels of high density lipoprotein (HDL). High TC was defined as a value >200 mg/dL and hypertriglyceridaemia as a serum TG >150 mg/dL. High LDL was defined as a serum LDL >135 mg/dL, while low HDL was defined as a serum HDL <40 mg/dL.^14^ Using the WHO criteria, a diagnosis of diabetes was made if the fasting blood glucose of the patient is ≥7.0 mmol/L or a 2-hour post-prandial glucose ≥11.1 mmol/L after a 75 g oral glucose load.^15^ A serum creatinine level >1.3 mg/dL was used to diagnose impairment in kidney function.^16^

### Sample size calculation

Considering the incidence of persistent hypertension of 49.9% and 25% for early onset versus late onset pre-eclampsia as found in a previous study^17^ and using the formula for cohort study,^14^ a sample size of 44 women with pre-eclampsia was calculated and this gives the study a statistical power of 80%, at 95% level of confidence, considering finite population correction factor,^18^ an attrition rate of 20% for loss to follow-up and other contingencies. The finite population correction factor,^19^ nf, was determined using the formula: nf = [(N-n)/(N-1)]1/2^1/2^, where: N = population size and n = sample size calculated. Findings obtained from scrutiny of previous records in the three study sites showed that 360 women with pre-eclampsia will be managed in the hospitals during the study period. Therefore, if N is 360 and n was calculated to be 38, substituting in the formula, nf = [(360 - 38)/(360 - 1)]1/2^1/2^ = 0.947.

### Sampling method

Eligible participants were enrolled by the consecutive sampling technique. The participants were recruited from the antenatal clinics, antenatal wards, emergency units and labour wards of the hospitals after obtaining informed consent.

### Data collection

Information was collected by direct questioning and from the case notes of each participant using a semistructured proforma, which included the sociodemographic characteristics, parity, last menstrual period, estimated gestational age at diagnosis of pre-eclampsia/ admission and expected date of delivery. The medical history, obstetric history, systolic and diastolic BP on admission and treatment regimens received were noted. Use of other medications in the ongoing pregnancy apart from the routine medications (iron, folic acid and vitamin C) was recorded. Pregnancy outcome was obtained by extracting information from the antenatal, labour, delivery and neonatal records after delivery. Foetal outcome measures such as the birth weight, the existence of intrauterine growth restriction and intrauterine demise were recorded. The phone numbers of each participant and an alternative phone number (for instance, that of her spouse) were collected before discharge so that the participant could be reached to remind her of her appointment. This was a strategy to minimise loss to follow-up in this research.

The participants were monitored from recruitment to delivery. Data were collected at three time points—enrolment at diagnosis of pre-eclampsia, delivery and at 6 weeks post partum. The parameters measured on admission included signs and symptoms to ascertain disease severity, BP measurement, urinalysis and urine output. Participants were asked questions about symptoms of severity to classify severe pre-eclampsia. These included headaches, blurring of vision, nausea, vomiting, epigastric and/or right-sided abdominal pain. They were also examined for signs like epigastric or right hypochondriac tenderness and ankle clonus.

BP was measured using the OMRON (HEM 9210T) digital BP monitor which is clinically validated for use in pregnancy. The same type of digital BP monitor was used in all the centres to prevent instrument bias. BP was checked using the left upper arm. The participant was in a semirecumbent position when the BP was taken. BP of 140/90–159/109 mm Hg at least 4 hours apart was classified as mild-moderate, while ≥160/110 mm Hg was classified as severe hypertension.

Urinalysis was done with the aid of urine test strips (Combi-10) when the participant voided, and the hourly urine output produced by the participant was measured and charted.

Participants were followed up till the end of the puerperium, which is 6 weeks post partum, when BP was rechecked, and blood samples for 75 g oral glucose tolerance test, fasting lipid profile and serum creatinine were obtained. BP was taken after the participant had rested for 15–30 min.

The participants were instructed to come in fasted (not eating or drinking anything, except water, within the 8–12 hours preceding their presentation for the 6 weeks post natal visit. At this visit, each participant first had their blood specimen collected by venepuncture for fasting blood glucose, fasting lipid profile and serum creatinine. 3 mL of blood was collected into each specimen bottle. Thereafter, a glucose drink was given to the patient to take slowly but within 5 min. The glucose drink was prepared by dissolving 75 g of anhydrous glucose in 250 mL of clean drinking water. Blood specimen was collected after 2 hours from commencement of the glucose drink for the 2-hour blood glucose assay to complete the oral glucose tolerance test (OGTT). The blood specimen for glucose assay was collected in vacutainer tubes containing fluoride oxalate. Blood specimen for serum creatinine was collected in plain vacutainer tubes. Blood specimen for fasting lipid profile was collected in vacutainers containing lithium heparin. All specimens were labelled with codes assigned to each patient. The samples were stored at 2–80°C in a refrigerator and transported within 1–48 hours of collection to the central research laboratory. This was to accommodate the weekends and closing hours of the laboratory.

Serum levels of fasting lipid profile and serum creatinine were determined using the Roche/Hitachi Cobas autoanalyser. Assay was performed at room temperature, and all reagents and samples were brought to room temperature. Samples were centrifuged at a speed of at least 1600 rcf for 10 min to obtain serum. This was done by the principal investigator in collaboration with the laboratory scientist. The decanted serum was placed in the sample compartment of the analyser and processed. Each of the analytes had a specific reagent cassette on the autoanalyser, which was inserted into the reagent compartment of the autoanalyser and was used to run the tests as the user programmed the machine. The Cobas Integra cassette reagent was used to analyse the samples. The autoanalyser was also used to analyse the samples of the blood glucose. All laboratory specimens were sent to the laboratory deidentified and with codes assigned.

Serum levels of fasting lipid profile and serum creatinine were determined using the Roche/ Hitachi Cobas autoanalyser. Assay was performed at room temperature and all reagents and samples were brought to room temperature. Samples were centrifuged at a speed of at least 1600 rcf for 10 min to obtain serum. This was done by the principal investigator in collaboration with the laboratory scientist. The decanted serum was placed in the sample compartment of the analyser and processed. Each of the analytes had a specific reagent cassette on the autoanalyser, which was inserted into the reagent compartment of the autoanalyser and was used to run the tests as the user programmed the machine. The Cobas Integra cassette reagent was used to analyse the samples. The autoanalyser was also used to analyse the samples of the blood glucose. All laboratory specimens were sent to the laboratory deidentified and with codes assigned.

### Quality control measures

Participant samples were stored at 2–8°C after collection, during transportation and before centrifugation using refrigerator and ice packs for transport. Samples were brought to room temperature before performing the assay. The materials included in the kit were quality-controlled materials. It was ensured that for every group of tests performed, the values of the concentrations lie within the limits stated for the relevant test kit lot. A quality control certificate containing these reference values was included in the kit. Where the values specified for the controls were not achieved, the test results were declared inaccurate, and the test was repeated.

### Exposure/ outcome measures

The exposure was pre-eclampsia defined for this study as new onset hypertension after 20 weeks gestation with significant proteinuria (2+albumin on dipstick testing).^20^ The outcomes were cardiovascular disease risk markers, viz persistent hypertension, glucose intolerance and dyslipidaemia and renal function at 6 weeks post partum in women who had pre-eclampsia. Hypertension was categorised as normal (BP not exceeding 120/80 mm Hg), prehypertension (systolic BP between 121 mm Hg and 139 mm Hg and/or diastolic BP between 81 mm Hg and 89 mm Hg) or hypertension (BP above 140/90 mm Hg).^21^ Glucose intolerance was defined as blood glucose levels above normal; fasting blood sugar level of 7.0 mmol/L and above and/or 2-hour postprandial glucose level of 11.0 mmol/L and above.^22^ Dyslipidaemia is defined as an abnormal value for any of the components of the fasting lipid profile.^23^ Renal function was assessed using serum creatinine levels only and defined as serum creatinine above 1.3 mg/dL based on the laboratory reference range.

### Statistical analysis

All data collected were subjected to statistical analysis using Stata V.16.1 (Stata Corp, College Station, Texas, USA). Descriptive statistics were computed. All continuous variables were presented as mean and SD. Discrete variables were presented as median and IQR. Categorical variables were presented as frequency and percentage. The proportion of women with early onset pre-eclampsia (defined as pre-eclampsia occurring before 34 weeks) and late onset pre-eclampsia (defined as pre-eclampsia occurring at 34 weeks and above), type of pre-eclampsia (mild vs severe pre-eclampsia) and proportion of pre-eclamptic women who remain at risk of cardiovascular disease (persistent hypertension, deranged blood glucose level, dyslipidaemia and abnormal renal function) were presented as percentages. Normality of the study population was ascertained using the Shapiro-Wilk test. Fisher’s exact test was used to ascertain if there is an association between time of onset of pre-eclampsia (early and late onset) and presence of persistent hypertension, dyslipidaemia, deranged blood glucose and abnormal renal function at the end of puerperium. OR with 95% CI was calculated to ascertain the magnitude of the effect of time of onset of pre-eclampsia (early vs late onset) on the presence of at least one cardiovascular disease risk factor (persistent hypertension, dyslipidaemia, deranged blood glucose or abnormal renal function) at 6 weeks post delivery. Adjustment in OR was made using the Cochran-Mantel-Haenszel method to cater for confounders such as age and body mass index (BMI) after stratifying these as age <40 years and age ≥40 years and BMI <30 kg/m^2^ and BMI ≥30 kg/m^2^, respectively, bearing in mind that age ≥40 years and BMI ≥30 kg/m^2^ (obesity) are independent risk factors for developing cardiovascular diseases. A post hoc analysis was done to determine the statistical power of this study in detecting a difference in the incidence of the outcome variables in women with early versus late onset pre-eclampsia. There was no missing data identified. The statistical significance level was set at p value <0.05 using two-tailed hypothesis.

### Patient and public involvement statement

Patients were not involved in the design and planning of the study.

## Result

A total of 44 eligible participants were studied, of whom 27 (61.4%) had mild pre-eclampsia and 17 (38.6%) had severe pre-eclampsia. The mean age±SD of all participants was 33.0±5.2 years and median parity 0 (IQR: 0–2). Women with late onset pre-eclampsia were significantly older than women with early onset pre-eclampsia (p<0.001). The median parity was 1 (IQR: 0–4) and the mean gestational age at recruitment was 29.9±6.5 weeks. The proportion of women with severe pre-eclampsia was higher in women who had early onset compared with late onset disease, but the difference was not statistically significant (p=0.507). All the women with early onset pre-eclampsia did not receive antenatal care at the facilities, while approximately 50% of women with late onset pre-eclampsia had antenatal care at the study sites. Table 1 shows details of the sociodemographic and clinical profile of the study population.

**Table 1:** Sociodemographic and clinical profile of women with pre-eclampsia

Characteristics	Total (n=44)	Early onset (n=13)	Late onset (n=31)
	Mean±SD	Mean±SD	Mean±SD
Maternal age (years)*	33.0±5.2	30.4±4.6	34.1±5.1
Mean gestational age at onset of pre-eclampsia (weeks)*	34.2±2.58	30.8±1.57	35.6±12.6
Mean body mass index (kg/m2^2^)†	24.4±0.7	25.9±1.46	23.8±0.77
Mean fetal birth weight (kg)*	4.520±0.986	3.647±0.690	4.886±0.856
Mean fetoplacental weight ratio*	4.52±0.99	3.65±0.69	4.89±0.86
	**Frequency (%)**	**Frequency (%)**	**Frequency (%)**
Severity of pre-eclampsia‡			
Mild	27 (61.4)	7 (53.8)	20 (64.5)
Severe	17 (38.6)	6 (46.2)	11 (35.5)
Status of antenatal care§			
Did not receive antenatal care	29 (65.9)	13 (100.0)	16 (51.6)
Received antenatal care	15 (34.1)	0 (0.0)	15 (48.4)
Marital status§			
Single	1 (2.3)	1 (7.7)	0 (0.0)
Married	43 (97.7)	12 (92.3)	31 (100.0)
Educational status§			
Primary	2 (4.5)	1 (7.7)	1 (3.2)
Secondary	12 (27.3)	1 (7.7)	11 (35.5)
Tertiary	30 (68.2)	11 (84.6)	19 (61.3)
Socioeconomic class§			
Low	15 (34.1)	2 (15.4)	13 (41.9)
Middle	26 (59.1)	10 (76.9)	16 (51.6)
High	3 (6.8)	1 (7.7)	2 (6.5)
Mode of delivery§			
Vaginal delivery	1 (2.3)	1 (7.7)	0 (0.0)
Caesarean section	43 (97.7)	12 (92.3)	31 (100.0)
Foetal survival‡			
Alive	39 (88.6)	8 (61.5)	31 (100.0)
Stillbirth	2 (6.8)	3 (15.4)	0 (0.0)
Early neonatal death	3 (4.6)	2 (23.1)	0 (0.0)

Categorical variables are presented as frequency (percentage) while continuous variables are presented as mean±SD.

P value <0.05 considered significant using two-tailed hypothesis.

* Student t-test.

† Mann Whitney U (two-sample Wilcoxon rank sum test).

‡χ 2 test used as test of hypothesis.

§ Fisher’s exact.

**Figure 1:**
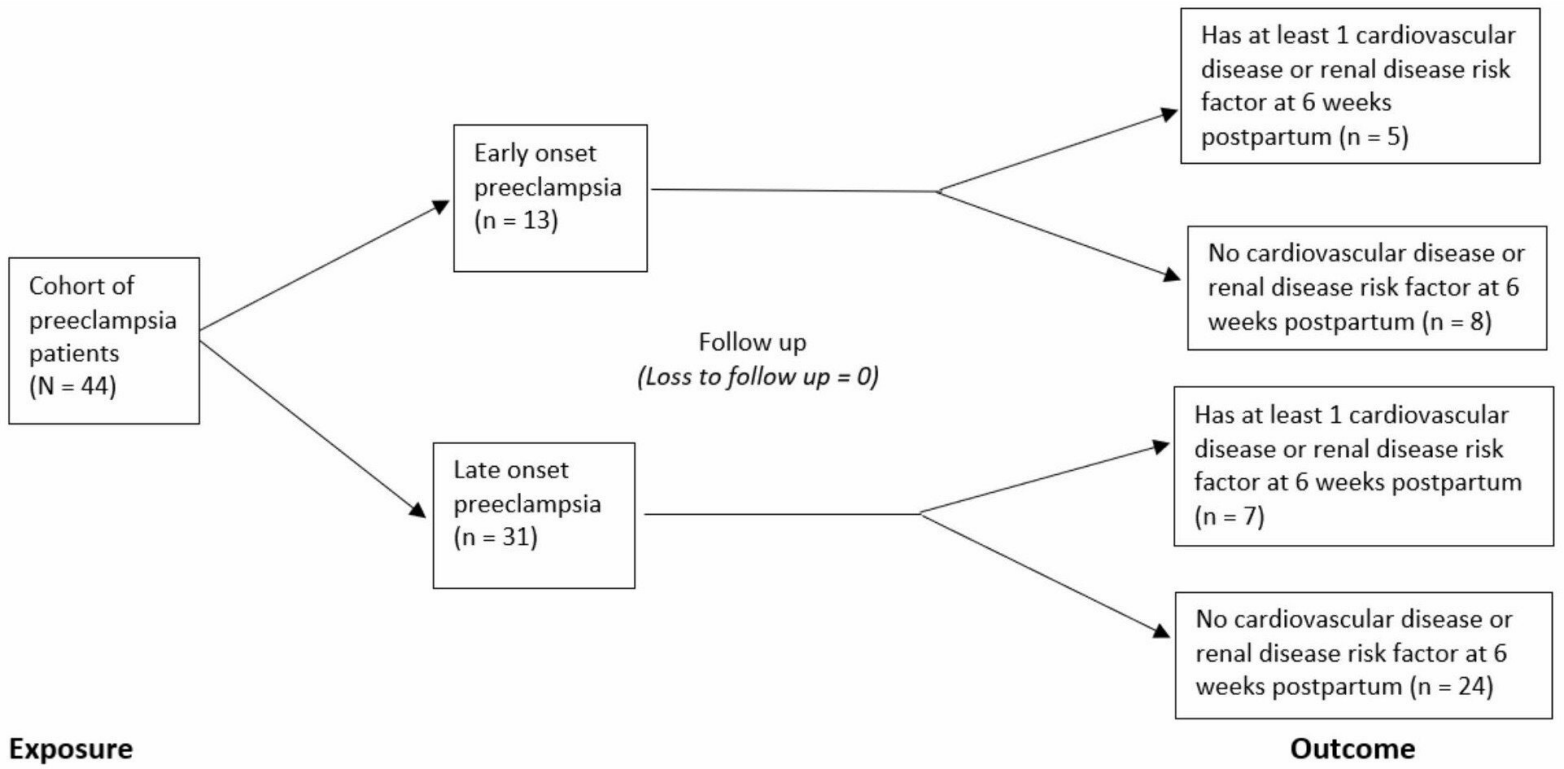
Flow diagram for cohort study.

Early onset pre-eclampsia was observed in 13 (29.5%) women while 31 (70.5%) women had late onset pre-eclampsia. The mean gestational age at diagnosis was 30.8±1.57 weeks in women with early onset pre-eclampsia and 35.6±1.26 weeks in women with late onset pre-eclampsia (p<0.001). figure 1 is a flow chart summarising the cohort study.

### Pre-eclampsia and cardiovascular disease risk at 6 weeks post partum

Of the cardiovascular disease risk markers, only serum TG concentration was found to be significantly higher in women with early onset pre-eclampsia compared with those with late onset pre-eclampsia, median values of 135 (IQR: 130–182) mg/dL vs 128 (IQR: 121–139) mg/dL, respectively (p=0.008). The mean TG levels were, however, all within normal limits. There was no difference in the mean or median levels of the other cardiovascular disease markers in both groups (p>0.05). table 2 summarises these findings.

**Table 2:** Mean values of cardiovascular and renal disease risk markers in women with early and late onset pre-eclampsia

Cardiovascular and renal disease risk marker	Early onset (n=13)	Late onset (n=31)	P value
Blood pressure readings			
Systolic blood pressure (mm Hg)**	132.1±11.2	128.3±9.6	0.263
Diastolic blood pressure (mm Hg)**	80.1±8.9	80.5±6.3	0.842
Mean arterial blood pressure (mm Hg)**	97.4±9.1	96.5±6.4	0.694
Oral glucose tolerance test			
Mean fasting blood sugar (mmol/L)**	5.37±0.47	5.10±0.31	0.164
Median 2-hour postprandial (mmol/L)†	7.1 (IQR: 6.8–7.8)	7.0 (IQR 6.8–7.3)	0.430
Fasting lipid profile			
Median high-density lipoprotein (mg/dL)†	65 (IQR: 61–69)	69 (IQR: 65–71)	0.430
Median low-density lipoprotein (mg/dL)†	121 (IQR: 116–124)	118 (IQR: 110–124)	0.273
Median serum cholesterol (mg/dL)†	141 (IQR: 130–172)	132 (IQR: 128–144)	0.212
Median triglycerides (mg/dL)†	135 (IQR: 130–182)	128 (IQR: 121–139)	0.008
**Median serum creatinine (µmol/L**)†	66.3 (IQR: 59.1–67.6)	63.8 (IQR: 60.6–66.7)	0.767

* Two sample student t-test, with equal variance.

† Two-sample Wilcoxon rank-sum (Mann-Whitney) test. p<0.05 considered significant using two-tailed hypothesis.

### Association between time of onset of pre-eclampsia (early and late pre-eclampsia) and risk of cardiovascular disease and renal impairment at 6 weeks post partum

In women with early onset pre-eclampsia, one (7.69%) had deranged OGTT, two (15.38%) had elevated serum cholesterol and LDL, three (23.08%) had low HDL, five (38.46%) had elevated TG levels and one (7.69%) had persistent hypertension. Among women who had late onset pre-eclampsia during pregnancy, none had derangement in OGTT, serum cholesterol and LDL values. One (3.23%) had low HDL, four (12.9%) had elevated TG levels and two (6.45%) had persistent hypertension. There was no statistically significant difference in the concentration of each biochemical parameter and BP levels in women with early versus late onset pre-eclampsia (p<0.05). Of the whole cohort of women with pre-eclampsia, eight (18.2%) became normotensive by 6 weeks post partum, while a large proportion of women, 33 (75.0%), had prehypertension. None of the pre-eclamptic women in both groups (early and late onset pre-eclampsia) had abnormal renal function at the end of puerperium. Table 3 summarises these findings.

**Table 3:** Cardiovascular and renal disease risk at 6 weeks post partum in women with early versus late onset pre-eclampsia

Cardiovascular and renal disease risk marker	Total(n=44)	Early onset(n=13)	Late onset(n=31)	Risk ratio	95% CI	P value
Blood pressure						
Normal	8 (18.2)	2 (15.4)	6 (19.4)	1.05	0.786 to 1.401	>0.999
Prehypertension	33 (75.0)	10 (76.9)	23 (74.2)			
Hypertension	3 (6.8)	1 (7.7)	2 (6.5)			
Oral glucose tolerance test						
Normal (fasting <7.0 mmol/L + 2 hours post-prandial <11.0 mmol/L)	43 (97.7)	12 (92.3)	31 (100.0)	–	–	0.295
Abnormal (fasting ≥7.0 mmol/L ± 2 HrPP ≥11.0 mmol/L)	1 (2.3)	1 (7.7)	0 (0.0)			
**Fasting lipid profile**						
High density lipoprotein						
Normal (>15 mg/dL)	40 (90.9)	10 (76.9)	30 (96.8)	7.15	0.818 to 62.563	0.071
Abnormal (<15 mg/dL)	4 (9.1)	3 (23.1)	1 (3.2)			
Low density lipoprotein						
Normal (<130 mg/dL)	42 (95.5)	11 (84.6)	31 (100.0)	–	–	0.082
Abnormal (>130 mg/dL)	2 (4.5)	2 (15.4)	0 (0.0)			
Cholesterol						
Normal (<200 mg/dL)	42 (95.5)	11 (84.6)	31 (100.0)	–	–	0.082
Abnormal (>200 mg/dL)	2 (4.5)	2 (15.4)	0 (0.0)			
Triglycerides						
Normal (<150 mg/dL)	35 (79.5)	8 (61.5)	27 (87.1)	2.98	0.949 to 9.359	0.098
Abnormal (>150 mg/dL)	9 (20.5)	5 (38.5)	4 (12.9)			
**Serum creatinine**						
Normal (<1.3 mg/dL)	44 (100.0)	13 (100.0)	31 (100.0)	–	–	–
Abnormal (>1.3 mg/dL)	0 (0.0)	0 (0.0)	0 (0.0)			

Figures represent proportions of women in each group presented as frequency (percentages). Fisher’s exact test was used to test for statistical significance for all variables. The relative risk, CIs and p values reported for hypertension compared the risk in early-onset versus late-onset pre-eclampsia with abnormal (prehypertension and hypertension combined) versus normal BP at 6 weeks postpartum.

Next, we evaluated the effect of age and BMI as confounders and effect modifiers of cardiovascular disease risk and renal function at 6 weeks post partum in women with early versus late pre-eclampsia.

Age and BMI did not have effect modification on cardiovascular disease and renal disease risk at 6 weeks postpartum in women with early and late onset pre-eclampsia. Age might, however, have some confounding effect on fasting lipid profile at 6 weeks post partum, especially for plasma lipoproteins and cholesterol (p<0.05). Table 4 gives details of this association.

**Table 4:** Effect modification and confounding on cardiovascular and renal disease risk at 6 weeks post partum in women with early versus late onset pre-eclampsia

Confounder/effect modifier	Crude OR (95% CI)	Adjusted OR (95% CI)	Breslow day p value	Mantel Haenszel p value
Age				
Persistent hypertension	0.828 (0.040 to 2.841)	0.577 (0.038 to 8.672)	0.103	0.664
Oral glucose tolerance test	–	–	>0.999	0.134
HDL	0.111 (0.002 to 1.654)	0.115 (0.011 to 1.255)	>0.999	0.046
LDL	–	–	>0.999	0.032
Serum cholesterol level	–	–	>0.999	0.032
Triglyceride level	0.237 (0.038 to 1.443)	0.243 (0.051 to 1.163)	>0.999	0.070
Serum creatinine	–	–	>0.999	–
Body mass index				
Persistent hypertension	0.828 (0.397 to 52.841)	5.513 (0.159 to 191.601)	0.773	0.366
Oral glucose tolerance test	–	–	>0.999	0.527
HDL	0.111 (0.002 to 1.654)	0.252 (0.015 to 4.264)	0.184	0.399
LDL	–	–	>0.999	0.327
Serum cholesterol level	–	–	>0.999	0.327
Triglyceride level	0.237 (0.038 to 1.444)	0.908 (0.087 to 9.472)	0.055	0.937
Serum creatinine	–	–	>0.999	–

HDL, high density lipoprotein; LDL, low density lipoprotein.

Breslow Day test was used in assessing effect modification, and Cochran Mantel Haenszel’s technique was used in testing for the confounding effect.

### Foetal outcome in early onset versus late onset pre-eclampsia

Women with late onset pre-eclampsia had a better fetal survival rate compared with women with early onset pre-eclampsia (p=0.001). The fetal birth weight is significantly higher in women with late onset pre-eclampsia compared with women with early onset pre-eclampsia (p≤0.001). Women with late onset pre-eclampsia had a significantly higher fetoplacental weight ratio than those with early disease (p≤0.001). These findings are as expected. Findings as they relate to foetal outcomes are presented in table 1.

### Post hoc analysis for power determination

In view of the lack of statistical significance for all outcome variables, we conducted a post-hoc power calculation to determine the statistical power of this study in detecting a difference in each cardiovascular disease risk marker in women with early onset pre-eclampsia and those with late onset preeclampsia. The sample size in this study provides a statistical power of 6.0% to detect a difference in the incidence of persistent hypertension at 6 weeks post partum between the women with early versus late onset pre-eclampsia at the 95% confidence level. For other outcomes, this study confers a statistical power of 40.5% to detect a difference in the incidence of abnormal glucose intolerance, 54.3% to detect a difference in the incidence of abnormal HDL, 57.5% to detect a difference in the incidence of abnormal LDL, 57.7% to detect a difference in the incidence of abnormal serum cholesterol level and 48.6% to detect a difference in the incidence of abnormal serum TG level at 6 weeks post partum at 95% confidence level.

## Discussion

The findings from this study suggest that women with late-onset pre-eclampsia constitute more than two-thirds of the participants. This higher proportion of late-onset pre-eclamptic women in this study agrees with the findings of other studies conducted in the USA and Poland, which also found a higher prevalence of late-onset pre-eclampsia.^24^,^26^ This is, however, contradictory to a higher incidence of early-onset pre-eclampsia found in the Mauritius population.^27^ The higher incidence of early-onset pre-eclampsia found in the Mauritius population is believed to be due to the high rate of preexisting chronic hypertension in this group of patients. A higher proportion of women with early-onset pre-eclampsia in this study had severe pre-eclampsia, similar to findings in a Polish study.^25^ Like outcomes from previous studies, our study showed that early-onset pre-eclampsia conferred a substantially higher risk for adverse foetal outcome than late-onset pre-eclampsia.^24^,^26^ This is related to earlier delivery in the mother with preterm babies predisposed to higher risk of morbidity and mortality.

Though previous studies have revealed significantly higher fasting blood sugar in women with early onset pre-eclampsia compared with women with late onset pre-eclampsia, this study did not find a statistically significant difference in mean values of blood glucose concentration in both groups.^17 28^ The level of TGs at 6 weeks post partum was higher in women with early onset pre-eclampsia compared with those with late onset pre-eclampsia, as seen in earlier studies.^17 28 29^ Whether our duration of follow-up in this study is adequate remains debatable. A recent systematic review and meta-analysis found that cardiovascular disease risk is doubled in pre-eclamptic compared with normotensive pregnant women and that this risk becomes noticeable at 1–3 years post-delivery and persists for close to 40 years afterwards.^30^ Dyslipidaemia in early onset pre-eclampsia had been found to persist till 3 months postpartum.^17^ Dyslipidaemia has been linked to endothelial cell dysfunction similar to what occurs in pre-eclampsia. However, the mechanism is not clear as to how pre-eclampsia or endothelial damage predisposes to dyslipidaemia.^26^

Three-quarters of the pre-eclamptic women studied had prehypertension, defined as systolic BP 121–139 mm Hg and/or diastolic value of 81–89 mm Hg,^21^ which still puts them at higher risk of having cardiovascular disease in the future. Several studies conducted earlier found significant numbers of persistent hypertension beyond the puerperium in patients with a history of pre-eclampsia.^11 17 28 29 31 32^ It has been shown that women with early-onset pre-eclampsia show high total vascular resistance compared with women with late-onset pre-eclampsia 1 year post partum.^33^ The loss of statistical significance in this study is likely because of the small sample size. It would be beneficial to explore this association in a larger, multicentre study as the occurrence of prehypertension or persistent hypertension at the end of puerperium might be an early warning sign and could also be a possible pathway for the development of chronic hypertension.

None of the participants in this study had abnormal renal function, as determined by their serum creatinine level 6 weeks post partum. Comparing women with early-onset pre-eclampsia to those with late-onset pre-eclampsia, there was no difference between the two groups in terms of adverse renal conditions and severe renal complications. This finding is supported by a cohort study in which about a quarter of pre-eclamptic patients with acute renal failure at delivery recovered completely within 6 weeks, as has been previously reported in other countries like Cameroon, America and the UK.^34^,^36^ Consistently, studies that have followed up women with hypertensive disorders during pregnancy including pre-eclampsia beyond 6 weeks post delivery have found some risk for cardiovascular disease beyond this period.^37^,^40^ A previous study in Uganda found one-third of pre-eclamptic women to have persistent hypertension at 3 months post delivery.^39^ A similar pattern was observed in Cameroon when pre-eclamptic women were followed up for 6 months; one-third of the women had persistent hypertension at this time point.^40^ This has an implication for practice in that there is a need for longer-term follow-up of all pregnant women with pre-eclampsia beyond the puerperium.

Age and BMI also had no effect modification on cardiovascular disease and renal disease risk at 6 weeks postpartum in women with early and late onset pre-eclampsia in this study. This study helped us identify the key cardiovascular risk markers that should be focused on (BP and fasting lipid profile) where funds are limited, thus minimising the financial burden of healthcare on the women and their families. Future studies should evaluate the cardiovascular risk markers at baseline and endpoint. Our participants did not have first trimester screening for pre-eclampsia. Though they were mostly unbooked women who did not have antenatal care at our facility, we routinely do not screen pregnant women for pre-eclampsia. It is thus difficult to ascertain the baseline risks of most of the women in our sample since they presented to the health facilities for the first time ill and with pre-eclampsia. Not assessing the baseline risk to detect women with pre-existing asymptomatic cardiovascular or renal disease is a limitation of this study. Only hypertension was assessed at baseline in this study, and this was only for the few women who had antenatal care at the facilities.

Recall bias could be a limitation in determining gestational age from last menstrual period in this study because some women may have irregular cycles and actual dates may sometimes be confusing if there is implantation bleeding. We would have preferred dating the pregnancy using crown-rump length obtained from an early scan done in the first trimester. However, many women register late for antenatal care in our setting, and most cases of pre-eclampsia presenting at our facility are unbooked patients who come in as emergency, that is, they did not receive antenatal care. In addition, they often do not have an early scan report.

Though this study is low in power to detect a difference in the incidence of the cardiovascular risk markers assessed in women with early versus late onset pre-eclampsia, it provides preliminary data for appropriate sample size calculation in future research. The low statistical power of this study is due to the use of data obtained from a high-income country, The Netherlands, for sample size calculation. The risk for cardiovascular disease differs by region, with a higher burden of cardiovascular disease in the low-income and middle-income countries compared with the high-income countries.^41^ This provides an explanation on why our study was underpowered, as we did not find appropriate local data from Nigeria or sub-Saharan Africa for sample size calculation when we designed the study.

Apart from the small sample size, another limitation of this study is that we did not collect data on antihypertensive use to determine if BP control is optimal. It will be beneficial in future research to adjust for antihypertensive use. In addition, doing a spot urine protein/creatinine ratio would have facilitated the identification of a few more cases of pre-eclampsia, as dipstick testing alone might not be sensitive enough. Patients with non-proteinuric pre-eclampsia were not captured in this study. Performing a 24-hour ambulatory BP monitor would have been best for a study of this nature, but we do not have the facility. This would have made it possible to pick up cases of transient hypertension and offer closer monitoring of the women. Having a baseline assessment of the women, preferably at booking and before the development of pre-eclampsia, will be valuable in future research of this nature.

It has also shown us that it is of utmost importance to closely monitor the BP of women with pre-eclampsia beyond the usual 6 weeks postpartum period, at the end of which they are routinely discharged from the postnatal clinics in Nigeria. However, how long should they be followed up remains a debatable question, and this opens ground for further longitudinal research to explore the duration of time that their risk for cardiovascular disease persists after an episode of pre-eclampsia. This will facilitate the modification of pre-existing protocols with regard to the management of women with pre-eclampsia in Nigeria.

### Conclusion

Serum TG concentration was significantly higher in early onset pre-eclamptic women compared with those with late onset disease. In addition, this study provides good cohort data which suggests that over 80% of pre-eclamptic women in Nigeria have prehypertension or persistent hypertension at 6 weeks post partum, indicating adverse cardiovascular remodelling and long-term risk of cardiovascular disease. This calls for a need for longer follow-up of these high-risk pregnant women beyond the puerperal period. The early detection of this cardiovascular disease risk marker will help in early diagnosis and prompt intervention, and thus help to stop or slow the progression of cardiovascular diseases. Larger longitudinal studies in diverse populations of pregnant women with pre-eclampsia, powered to determine the association between time of onset of pre-eclampsia and long-term cardiovascular risks, are necessary to further elucidate these findings.

## Data Availability

Data are available upon reasonable request.
