# Supplementary material for: Time of onset of pre-eclampsia as a determinant of risk of cardiovascular disease and renal impairment at six weeks post partum: a cohort study in Lagos, Nigeria
Source: BMJ Open. 2025 Jul 15;15(7):e094397. doi: 10.1136/bmjopen-2024-094397 (PMC12265814; doi:10.1136/bmjopen-2024-094397)
Supplement: Supplementary file 1 [file bmjopen-2024-094397.reviewer_comments.pdf]

PEER REVIEW HISTORY

BMJ Open publishes all reviews undertaken for accepted manuscripts. Reviewers are asked to complete a checklist review form and are provided with free text boxes to elaborate on their assessment. These free text comments are reproduced below.

ARTICLE DETAILS

Title (Provisional)

Time of onset of pre-eclampsia as a determinant of risk of cardiovascular disease and renal impairment at six weeks post-partum: a cohort study in Lagos, Nigeria

Authors

Kusamotu, Olaniyi Araotan; BABAH, OCHUWA Adiketu; Udenze, Ifeoma; Oluwole, Ayodeji A.; Afolabi, Bosede Bukola

---

VERSION 1 - REVIEW

**Reviewer**	**1**
**Name**	**Di Simone , Nicoletta**
**Affiliation**	**Humanitas University**
**Date**	**22-Dec-2024**
**COI**	**None**

The authors sought to perform an interesting study on the long term cardiovascular and renal risks in women who expenrienced preeclampsia. The manuscript is well written, just few suggestions for the discussion:

- can you please expand on the potential cardiovascular risks after one year post delivery as explained in this meta analysis : Inversetti A, Pivato CA, Cristodoro M, Latini AC, Condorelli G, Di Simone N, Stefanini G. Update on long-term cardiovascular risk after pre-eclampsia: a systematic review and meta-analysis. Eur Heart J Qual Care Clin Outcomes. 2024 Jan 12;10(1):4-13. doi: 10.1093/ehjqcco/qcad065. PMID: 37974053.

- can you please expand on whether the recruited patients underwent first trimester screening for preeclampsia or not?

**Reviewer**	**2**
**Name**	**Kitt, Jamie**
**Affiliation**	**University of Oxford, Department of Cardiovascular Medicine**
**Date**	**03-Jan-2025**
**COI**	**None**

In the abstract please outline how many women were discharge on anti-hypertensives and remained on treatment with anti-hypertensives at the 6 week point. I think the fact that only 18/18.2% were normotensive at 6 weeks highlights a massive under treatment of this cohort and this message really needs to come across in the discussion and conclusion. Please reword to strengthen this message

An additional limitation is the small sample size. Can you please provide the finite population correction factor details? With only 13 in the early onset PET the value of a p Value comparing the characteristics/demographics in table 1 is questionable. Certainly the BMI of late onset is usually higher in systematic reviews/larger sample sizes and is the opposite of what is seen here so this needs exploring in the discussion in lines 23-50.

In table 2 was adjustment made for booking blood pressures - if this data is available it should be include in table 1 and an adjusted difference calculated for table 2

Table 3 - please define and reference the BP values used in the classification of normal/pre-hypertension and hypertension e.g. most recent ESC or the most recent AHA guidance. Also include this in lines 20-33 of the discussion

In the discussion lines 20-33 discuss how ~75% still had pre-hypertension beyond the puerperium which is in keeping with several other studies as referenced. However the data here does not explain the discussion from lines 26-33 as the perecentage with prehypertension in early and late onset PET is the same in this study, as is the % with hypertension, so postulation about the role of PVR/TVR is early onset is not justified.

In lines 35-50 I would argue that perhaps we aren't using sensitive enough diagnostic tools - see earlier comment about urine ACR. Some studies have also showed differences in renal size and inflammation assessed using T1 maps in MRI scans (ref:

Hannah Rebecca Cutler, Logan Barr, Prenali Dwisthi Sattwika, Annabelle Frost, Mohanad Alkhodari, Jamie Kitt, Winok Lapidaire, Adam James Lewandowski, Paul Leeson, Temporal patterns of pre- and post-natal target organ damage associated with hypertensive pregnancy: a systematic review, European Journal of Preventive Cardiology, Volume 31, Issue 1, January 2024, Pages 77–99, https://doi.org/10.1093/eurjpc/zwad275)

Table 4 formatting needs adjusting as it's not as easy to follow at present

Other comment:

Is urine ACR not routinely available in the postpartum clinic these women are seen in - there is prior evidence that persistent of microalbuminuria at 6 weeks correlates to elevated long-term risk of CKD. Suggest including it in future work.

Is SFLT available in Nigeria - if not - ignore this comment - if so bring this into the discussion as several studies show correlation to long-term CV risk (ref: Angiogenic Factors and Long-Term Cardiovascular Risk in Women That Developed Preeclampsia During Pregnancy Carmen Garrido-Gimenez, Manel Mendoza et al. Hypertension

Volume 76, Number 6 https://doi.org/10.1161/HYPERTENSIONAHA.120.15830

**Reviewer**	**3**
**Name**	**Turé, Ruben**
**Affiliation**	**University of Porto Institute of Public Health, EPIunit**
**Date**	**17-Mar-2025**
**COI**	**None**

ABSTRACT

In the outcome measures section of the abstract, in page 3, line 3, provide a more precise definition of the exposure. The statement the "the exposure was pre-eclampsia" is too broad. It should clarify whether preeclampsia was classified as early-onset vs late-onset, and the diagnostic criteria used to define preeclampsia should also be explicitly stated. In the same paragraph, define each outcome with the clinical thresholds used. For instance, "The primary outcomes were cardiovascular disease risk markers, including persistent hypertension (BP ≥140/90 mmHg at 6 weeks postpartum), glucose intolerance (fasting glucose ≥5.6 mmol/L or 2-hour OGTT ≥7.8 mmol/L), dyslipidaemia (LDL ≥130 mg/dL, triglycerides ≥150 mg/dL), and renal dysfunction (serum creatinine ≥90 µmol/L or persistent proteinuria)."

INTRODUCTION

In page 4, the first paragraph and first line of the introduction section, the definition of preeclampsia appears to focus only on hypertension and proteinuria. However, updated guidelines from ISSHP, ACOG, and WHO also include cases of preeclampsia with end-organ dysfunction in the absence of proteinuria. The authors may wish to revise this section to reflect the broader diagnostic criteria now recognized in international guidelines.

In line 51 of page 4, while the sentence is a well-established concept in medical literature, it lacks a supporting reference. Given the importance of evidence-based writing, a citation from a peer-reviewed source should be added.

In line 13, of page 5, the statement highlights an important clinical gap in postpartum care in your setting, but lacks a supporting evidence. If no published data exists, consider specifying "In our clinical experience" or referencing local health policies or guidelines.

In page 5, line 34 (objectives), the study objectives are relevant and suggest a combination of descriptive and analytical aims, which may not align with the study's actual design and statistical power. Since the classification of early- vs. late-onset preeclampsia is already embedded in the study design, the first objective may not be necessary as a standalone aim. Instead, this information could be incorporated into the methods section. Similarly, the objectives related to determining the proportion of women with cardiovascular and renal risk markers at the end of the puerperium suggest a descriptive approach. However, if the study sample is not representative of the general population, the prevalence estimates derived from this study may not be generalizable. Since the primary value of this research lies in assessing whether the time of onset of preeclampsia is associated with cardiovascular and renal risk markers, the focus should be placed on this analytical objective rather than on descriptive estimates. Consider rewriting the objectives to focus on the association between time of onset

of pre-eclampsia (early versus late onset), and cardiovascular risk markers such as hypertension, dyslipidaemia, high blood glucose and renal function at the end of puerperium.

METHODS AND ANALYSIS

Study setting

In page 6, line 5 (study design and setting), The study identifies the hospitals where data was collected but does not provide details on why these sites were chosen. Including a brief justification, such as the hospitals' patient volume, referral status, or role in managing preeclampsia cases, would strengthen the methodological rigor by showing that the setting is appropriate for answering the research question.

Eligibility criteria

How are the authors defining preeclampsia for the study? Would someone with eclampsia without proteinuria be included? Would someone with new onset hypertension and HELLP syndrome without proteinuria be included? Would new onset hypertension with fetal growth restriction and no proteinuria be defined as having preeclampsia?

In page 6, line 17 (study population), the inclusion criteria could be expanded to improve clarity and ensure that the target population is well defined. Since the study investigates the impact of the time of onset of preeclampsia, specifying whether gestational age at diagnosis was used for stratification into early-onset (<34 weeks) and late-onset (≥34 weeks) preeclampsia would enhance the methodological transparency. Providing this detail would help ensure that differences between these groups are appropriately captured in the analysis.

In page 6, line 50, the reference 19 does not support the definition of severe preeclampsia. Reference 19 is: Anthony MD, Jaye PF. Lipids and the endothelium. Please use recent evidence for the definition of severe preeclampsia.

Data collection

In page 7, line 15, using the most recent ultrasound scan is a more reliable method, and it should be the primary approach for defining gestational age, with LMP used only if ultrasound is unavailable. Relying on the LMP alone can be inaccurate due to irregular cycles, recall bias, or implantation bleeding misinterpretation.

In page 7, line 23, the references cited in this paragraph do not support the definitions provided for dyslipidaemia, diabetes, or renal impairment. Proper referencing is crucial for ensuring scientific accuracy and credibility.

In page 7, line 40 (Sample size calculation), the sample size calculation appears to underestimate the required number of participants for adequate statistical power. Based on the assumptions provided, a correct calculation suggests at least 69 participants per group (138 total). If feasibility constraints limited recruitment, the authors should either (1) justify the smaller sample size and acknowledge its impact on power or (2) consider a post-hoc power analysis to assess the robustness of findings.

In page 9, line 12, the authors stated that participants were followed up until the end of the puerperium (6 weeks postpartum), but the frequency of follow-up visits during the postpartum period is not clearly described. If assessments were limited to a single visit at six weeks postpartum, this should be explicitly stated. If additional follow-ups were conducted, providing details on what was monitored and how often could strengthen the methodology section.

Outcomes

In page 10, line 32 (outcome measures), the exposure and outcome measures are not suffieciently detailed as mentioned in the abstract comments. Please define the outcome measures and provide evidence to support each definition.

In page 10, line 44, the authors mention qualitative variables being presented as frequency and percentage, but no qualitative variables were described in the study. Do the authors mean categorical variables? The definitions mentioned in the statistical analysis section (early vs late-onset preeclampsia) should remain in the data collection section, rather than being repeated.

DISCUSSION

In the discussion section, the article could benefit from contextualizing your findings by comparing them with existing studies on postpartum cardiovascular impairment in SSA. This comparison will highlight the relevance of your research and identify consistencies or discrepancies with regional data. These studies focus on postpartum cardiovascular risks among women with hypertensive disorders of pregnancy, including preeclampsia, in SSA:

- Postpartum trend in blood pressure levels, renal function and proteinuria in women with severe preeclampsia and eclampsia in Sub-Saharan Africa: A 6-months cohort study
- Hypertension Persisting after Pre-Eclampsia: A Prospective Cohort Study at Mulago Hospital, Uganda
- Persistent Hypertension after Preeclampsia in a Group of Cameroonians: Result of a Cross-Sectional Study and Perspectives to Reduce its Burden in Limited Income Countries
- Persistent Hypertension Up to One Year Postpartum among Women with Hypertensive Disorders in Pregnancy in a Low-Resource Setting: A Prospective Cohort Study
- Postpartum Metabolic Syndrome after Gestational Hypertension and Preeclampsia, a Prospective Cohort Study

The discussion frequently names authors directly (e.g., "Lisonkova S et al," "Weitzner O et al"), which is more typical of social sciences rather than epidemiological research. In clinical and public health studies, a numeric citation format (e.g., [17,18]) or an author-year system (e.g., Lisonkova et al., 2017) is generally preferred. The authors might consider adjusting the citation style for consistency with standard epidemiological writing.

The discussion would benefit from addressing whether six weeks postpartum is a sufficient timeframe to detect meaningful cardiovascular and renal dysfunction. Many studies follow women for a longer period, as postpartum hypertension and renal impairment may not be fully apparent at six weeks. Since some cases of hypertension resolve spontaneously while others persist and indicate long-term cardiovascular risk, discussing the potential need for extended follow-up would enhance the interpretation of the findings.

Another important aspect to clarify is whether the study differentiates between persistent hypertension and transient postpartum hypertension. Blood pressure fluctuations are common in the postpartum period, and it is unclear whether hypertension detected at six weeks was classified as chronic or if resolution was observed in some women. Differentiating between transient and persistent hypertension is crucial, as the long-term cardiovascular implications differ. Addressing this in the discussion would provide a clearer understanding of the clinical significance of the findings.

The accuracy of blood pressure measurement methods should also be acknowledged. The study relies on office blood pressure readings, but 24-hour ambulatory blood pressure monitoring is considered the gold standard for detecting true hypertension, particularly in distinguishing between white-coat hypertension and masked hypertension. If ambulatory monitoring was not used, discussing the potential for misclassification would add transparency and highlight a possible limitation.

The discussion should include a structured acknowledgment of all the key study limitations. The absence of baseline cardiovascular and renal function is an important limitation, but not the only one. I suggest the authors to consider addressing other important limitations such as the small sample size and its impact on statistical power.

LANGUAGE

I would also suggest a double check for English style and language.

VERSION 1 - AUTHOR RESPONSE

Reviewer 1

1. Can you please expand on the potential cardiovascular risks after one year post delivery as explained in this meta analysis : Inversetti A, Pivato CA, Cristodoro M, Latini AC, Condorelli G, Di Simone N, Stefanini G. Update on long-term cardiovascular risk after pre-eclampsia: a systematic review and meta-analysis. Eur Heart J Qual Care Clin Outcomes. 2024 Jan 12;10(1):4-13. doi: 10.1093/ehjqcco/qcad065. PMID: 37974053.

Response

Some expansions of the discussion have been made, reflecting on longer term cardiovascular risk that have been found in other study, highlighting the limitation of a shorter follow up period in our study which might explain why some of the cardiovascular risks were not evident. The addition reads thus: "Whether our duration of follow-up in this study is adequate remains debatable. A recent systematic review and metaanalysis found that cardiovascular disease risk is doubled in preeclamptic compared to normotensive pregnant women and that this risk becomes noticeable at one to three years post-delivery and persists for close to forty years afterwards [32]". Last five lines of the first paragraph of Page 18. Appropriate reference has also been cited.

Reference

32. Inversetti A, Pivato CA, Cristodoro M, Latini AC, Condorelli G, Di Simone N, et al. Update on long-term cardiovascular risk after pre-eclampsia: A systematic review and meta-analysis. European Heart Journal-Quality of Care and Clinical Outcomes. 2024;10(1):4-13.

2. Can you please expand on whether the recruited patients underwent first trimester screening for preeclampsia or not?

Response

Thank you for bringing this forth. The participants did not have a first trimester screening for preeclampsia. This is not widely practiced in our environment. We have included a sentence on this in the discussion. It reads thus: "Our participants did not have first trimester screening for preeclampsia. Though they were mostly unbooked women who did not have antenatal care at our facility, we routinely do not screen pregnant women for preeclampsia." Page 19, paragraph 2.

Reviewer 2

1. In the abstract, please outline how many women were discharge on anti- hypertensives and remained on treatment with anti-hypertensives at the 6 week point. I think the fact that only 18/18.2% were normotensive at 6 weeks highlights a massive under treatment of this cohort and this message really needs to come across in the discussion and conclusion. Please reword to strengthen this message. **Response**

Thank you for this question. Unfortunately, we did not collect data on antihypertensive use in the puerperium. What have added this as a limitation for this study and emphasize the possibility of sub-optimal treatment and that this gap needs to be filled in future research on similar subjects. It reads: "Another limitation of this study is that we did not collect data on antihypertensive use to determine if blood pressure control is optimal. It will be beneficial in future research to adjust for antihypertensive use". Page 20, paragraph 1.

2. An additional limitation is the small sample size. Can you please provide the finite population correction factor details? With only 13 in the early onset PET the value of a p Value comparing the characteristics/demographics in table 1 is questionable. Certainly, the BMI of late onset is usually higher in systematic reviews/larger sample sizes and is the opposite of what is seen here so this needs exploring in the discussion in lines 23-50.

Response

Thank you for these questions. We have provided details on the finite population correction factor. The additional note reads thus: "The finite population correction factor[51], nf was determined using the formula: $nf = \{(N-n)/(N-1)\}^{1/2}$, where: N = population size and n = sample size calculated. Findings obtained from scrutiny of previous records in the three (3) study sites showed that 360 women with preeclampsia will be managed in the hospitals during study period. Therefore, if N is 360 and n was calculated to be 38, substituting in the formula, $nf = \{(360 - 38)/(360 - 1)\}^{1/2} = 0.947$". Page 7, section on sample size. In view of sample size, we have thus removed p-values in Table 1, Page 12.

3. In table 2 was adjustment made for booking blood pressures - if this data is available it should be include in table 1 and an adjusted difference calculated for table 2. **Response**

Thank you. We did not make an adjustment for the blood pressure at booking because we do not have data on blood pressure at booking; more so 65.9% of the participants were unbooked women presenting for the first time with preeclampsia (Table 1).

4. Table 3 - please define and reference the BP values used in the classification of normal/pre-hypertension and hypertension e.g. most recent ESC or the most recent AHA guidance. Also include this in lines 20-33 of the discussion.

Response

Thank you. This has been done in the methods and discussion sections, Page 10, paragraph 2 and Page 18, paragraph 3 respectively.

5. In the discussion lines 20-33 discuss how ~75% still had pre-hypertension beyond the puerperium which is in keeping with several other studies as referenced. However the data here does not explain the discussion from lines 26-33 as the perecentage with

prehypertension in early and late onset PET is the same in this study, as is the % with hypertension, so postulation about the role of PVR/TVR is early onset is not justified.

Response

Thank you for this comment. We have re-written the affected section. It reads thus: "The loss of statistical significance in this study is likely because of small sample size. It would be beneficial to explore this association in a larger, multicentre study as the occurrence of prehypertension or persistent hypertension at the end of puerperium might be an early warning sign and could also be a possible pathway for the development of chronic hypertension." Page 18, paragraph 3.

6. In lines 35-50 I would argue that perhaps we aren't using sensitive enough diagnostic tools - see earlier comment about urine ACR. Some studies have also showed differences in renal size and inflammation assessed using T1 maps in MRI scans (ref: Hannah Rebecca Cutler, Logan Barr, Prenali Dwisthi Sattwika, Annabelle Frost, Mohanad Alkhodari, Jamie Kitt, Winok Lapidaire, Adam James Lewandowski, Paul Leeson, Temporal patterns of pre- and post-natal target organ damage associated with hypertensive pregnancy: a systematic review, European Journal of Preventive Cardiology, Volume 31, Issue 1, January 2024, Pages 77–99, https://doi.org/10.1093/eurjpc/zwad275).

Response

Thank you. We agree doing a spot urine protein/creatinine ratio would be facilitated the identification of a few more cases of preeclampsia as dipstick testing alone might not be sensitive enough. This has been added as a limitation of this study. Page 20, paragraph 1.

7. Table 4 formatting needs adjusting as it's not as easy to follow at present.

Response

Thank you for bringing this to our notice. We have re-formatted Table 4 to enhance clarity. Page 16.

8. Is urine ACR not routinely available in the postpartum clinic these women are seen in- there is prior evidence that persistent of microalbuminuria at 6 weeks correlates to elevated long-term risk of CKD. Suggest including it in future work. Is SFLT available in Nigeria - if not - ignore this comment - if so bring this into the discussion as several studies show correlation to long-term CV risk (ref: Angiogenic Factors and Long-Term Cardiovascular Risk in Women That Developed Preeclampsia During Pregnancy Carmen Garrido-Gimenez, Manel Mendoza et al. Hypertension Volume 76, Number 6 https://doi.org/10.1161/HYPERTENSIONAHA.120.15830 **Response**

Thank you. Urine albumin/creatinine ratio is not routinely done in our clinics. However, we may request it when there is a need to confirm significant proteinuria in preeclamptic patients with 1+ proteinuria on dipstick testing; the turnaround for getting result from the laboratory is sometimes discouraging. This has been added as a limitation of this study. Page 20, paragraph 1. sFLT is not readily available in Nigeria. We will consider these in future research.

Reviewer 3

1. Abstract - In the outcome measures section of the abstract, in page 3, line 3, provide a more precise definition of the exposure. The statement the "the exposure was pre-eclampsia" is too broad. It should clarify whether preeclampsia was classified as early-onset vs late-onset, and the diagnostic criteria used to define preeclampsia should also be

explicitly stated. In the same paragraph, define each outcome with the clinical thresholds used. For instance, “The primary outcomes were cardiovascular disease risk markers, including persistent hypertension (BP ≥140/90 mmHg at 6 weeks postpartum), glucose intolerance (fasting glucose ≥5.6 mmol/L or 2-hour OGTT ≥7.8 mmol/L), dyslipidaemia (LDL ≥130 mg/dL, triglycerides ≥150 mg/dL), and renal dysfunction (serum creatinine ≥90 µmol/L or persistent proteinuria).”

Response

Thank you for this question. This was a single arm cohort study, that is, the pregnant women with preeclampsia were enrolled irrespectively of whether they were early or late onset; segregation into these categories was made later and not as a criterion for enrolment. Preeclampsia we defined as new onset hypertension after 20 weeks gestation with significant proteinuria. The exposures and outcomes have now been defined in the section on exposures and outcomes on Page 10, paragraph 2.

2. Introduction **-** In page 4, the first paragraph and first line of the introduction section, the definition of preeclampsia appears to focus only on hypertension and proteinuria. However, updated guidelines from ISSHP, ACOG, and WHO also include cases of preeclampsia with end-organ dysfunction in the absence of proteinuria. The authors may wish to revise this section to reflect the broader diagnostic criteria now recognized in international guidelines.

Response

Thank you. This is well noted. The definition of preeclampsia in the introduction has now been updated and a new reference [1] added. Page 3, first paragraph of introduction.

3. In line 51 of page 4, while the sentence is a well-established concept in medical literature, it lacks a supporting reference. Given the importance of evidence-based writing, a citation from a peer-reviewed source should be added.

Response

Thank you. The sentence has been rephrased and a citation has been added. Page 4, paragraph 2.

4. In line 13, of page 5, the statement highlights an important clinical gap in postpartum care in your setting, but lacks a supporting evidence. If no published data exists, consider specifying "In our clinical experience" or referencing local health policies or guidelines.

Response

Thank you for this valuable suggestion. The sentence has been rephrased accordingly by specifying that it was based on local experience. It reads thus: “In our clinical experience, since majority of the pregnant women .... ” Page 5 under study population.

5. In page 5, line 34 (objectives), the study objectives are relevant and suggest a combination of descriptive and analytical aims, which may not align with the study's actual design and statistical power. Since the classification of early- vs. late-onset preeclampsia is already embedded in the study design, the first objective may not be necessary as a standalone aim. Instead, this information could be incorporated into the methods section. Similarly, the objectives related to determining the proportion of women with cardiovascular and renal risk markers at the end of the puerperium suggest a descriptive approach. However, if the study sample is not representative of the general population, the prevalence estimates derived from this study may not be generalizable. Since the primary value of this

research lies in assessing whether the time of onset of preeclampsia is associated with cardiovascular and renal risk markers, the focus should be placed on this analytical objective rather than on descriptive estimates. Consider rewriting the objectives to focus on the association between time of onset of pre-eclampsia (early versus late onset), and cardiovascular risk markers such as hypertension, dyslipidaemia, high blood glucose and renal function at the end of puerperium.

Response

Thank you for your suggestion. The objective has been rewritten as suggested. Page 4, last paragraph under objective.

6. Study setting **-** In page 6, line 5 (study design and setting), The study identifies the hospitals where data was collected but does not provide details on why these sites were chosen. Including a brief justification, such as the hospitals' patient volume, referral status, or role in managing preeclampsia cases, would strengthen the methodological rigor by showing that the setting is appropriate for answering the research question.

Response

Thank you for this suggestion. Additional notes have been made on study sites. Page 5, paragraph 1.

7. Eligibility criteria **-** How are the authors defining preeclampsia for the study? Would someone with eclampsia without proteinuria be included? Would someone with new onset hypertension and HELLP syndrome without proteinuria be included? Would new onset hypertension with fetal growth restriction and no proteinuria be defined as having preeclampsia?

Response

Thank you. Cases of preeclampsia without proteinuria are not common in our environment. More so, we do not want cases of gestational hypertension to be mistakenly included. Since we had as inclusion criteria hypertension with significant proteinuria, we have included as a limitation in the discussion the exclusion of cases of preeclampsia without proteinuria. Page 20, paragraph 1. In future research we will consider this category.

8. In page 6, line 17 (study population), the inclusion criteria could be expanded to improve clarity and ensure that the target population is well defined. Since the study investigates the impact of the time of onset of preeclampsia, specifying whether gestational age at diagnosis was used for stratification into early-onset (<34 weeks) and late-onset (≥34 weeks) preeclampsia would enhance the methodological transparency. Providing this detail would help ensure that differences between these groups are appropriately captured in the analysis.

Response

Thank you. We did not use gestational age as an inclusion criterion because it was a single arm cohort study. All preeclamptic who met the inclusion criteria were enrolled as a single cohort. The segregation based on gestational age was made afterwards. Page 5, second line of study population.

9. In page 6, line 50, the reference 19 does not support the definition of severe preeclampsia. Reference 19 is: Anthony MD, Jaye PF. Lipids and the endothelium. Please use recent evidence for the definition of severe preeclampsia.

Response

Thank you for drawing our attention to this. Reference 19 has been changed to a more appropriate reference defining preeclampsia with severe features. Page 6, paragraph 1; Reference 12.

10. Data collection **-** In page 7, line 15, using the most recent ultrasound scan is a more reliable method, and it should be the primary approach for defining gestational age, with LMP used only if ultrasound is unavailable. Relying on the LMP alone can be inaccurate due to irregular cycles, recall bias, or implantation bleeding misinterpretation.

Response

Thank you. We would have preferred the earliest scan preferably one done in the first trimester. However, many women register late for antenatal care. Furthermore, most cases of preeclampsia presenting at our facility are unbooked patients who come in as emergency, that is, they did not receive antenatal care. In addition, they often do not have an early scan report.

11. In page 7, line 23, the references cited in this paragraph do not support the definitions provided for dyslipidaemia, diabetes, or renal impairment. Proper referencing is crucial for ensuring scientific accuracy and credibility.

Response

Thank you. Affected reference has been reviewed and changed. Page 6, last paragraph.

12. In page 7, line 40 (Sample size calculation), the sample size calculation appears to underestimate the required number of participants for adequate statistical power. Based on the assumptions provided, a correct calculation suggests at least 69 participants per group (138 total). If feasibility constraints limited recruitment, the authors should either (1) justify the smaller sample size and acknowledge its impact on power or (2) consider a post-hoc power analysis to assess the robustness of findings.

Response

Thank you. Small sample size has been acknowledged as a limitation in the discussion. Page 18, paragraph 3. Additional information on the finite population correction factor has been added. Page 7, paragraph 1.

13. In page 9, line 12, the authors stated that participants were followed up until the end of the puerperium (6 weeks postpartum), but the frequency of follow-up visits during the postpartum period is not clearly described. If assessments were limited to a single visit at six weeks postpartum, this should be explicitly stated. If additional follow-ups were conducted, providing details on what was monitored and how often could strengthen the methodology section.

Response

Thank you. The participants were reassessed once postpartum at their six weeks postpartum visit. This has been clarified in the methodology. It reads: "Data was collected at three timepoints – enrolment at diagnosis of preeclampsia, delivery and at six weeks postpartum". Page 8, paragraph 2.

14. Outcomes - In page 10, line 32 (outcome measures), the exposure and outcome measures are not sufficiently detailed as mentioned in the abstract comments. Please define the outcome measures and provide evidence to support each definition.

Response

Thank you. Outcomes and exposures have been defined and references provided where applicable. Page 10, paragraph 2.

15. In page 10, line 44, the authors mention qualitative variables being presented as frequency and percentage, but no qualitative variables were described in the study. Do the authors mean categorical variables? The definitions mentioned in the statistical analysis section (early vs late- onset preeclampsia) should remain in the data collection section, rather than being repeated.

Response

Thank you. This is noted. Repetitions have been deleted. We prefer the term categorical variable to qualitative variables. Page 10, last paragraph.

16. Discussion - In the discussion section, the article could benefit from contextualizing your findings by comparing them with existing studies on postpartum cardiovascular impairment in SSA. This comparison will highlight the relevance of your research and identify consistencies or discrepancies with regional data. These studies focus on postpartum cardiovascular risks among women with hypertensive disorders of pregnancy, including preeclampsia, in SSA:

- Postpartum trend in blood pressure levels, renal function and proteinuria in women with severe preeclampsia and eclampsia in Sub-Saharan Africa: A 6-months cohort study
- Hypertension Persisting after Pre-Eclampsia: A Prospective Cohort Study at Mulago Hospital, Uganda
- Persistent Hypertension after Preeclampsia in a Group of Cameroonians: Result of a Cross-Sectional Study and Perspectives to Reduce its Burden in Limited Income Countries
- Persistent Hypertension Up to One Year Postpartum among Women with Hypertensive Disorders in Pregnancy in a Low-Resource Setting: A Prospective Cohort Study
- Postpartum Metabolic Syndrome after Gestational Hypertension and Preeclampsia, a Prospective Cohort Study

Response

Thank you for your suggestions. We have added additional notes in the discussion to make regional comparisons. Page 19, paragraph 1.

17. The discussion frequently names authors directly (e.g., "Lisonkova S et al," "Weitzner O et al"), which is more typical of social sciences rather than epidemiological research. In clinical and public health studies, a numeric citation format (e.g., [17,18]) or an author-year system (e.g., Lisonkova et al., 2017) is generally preferred. The authors might consider adjusting the citation style for consistency with standard epidemiological writing.

Response

Thank you. Citation style has been adjusted all through the manuscript.

18. The discussion would benefit from addressing whether six weeks postpartum is a sufficient

timeframe to detect meaningful cardiovascular and renal dysfunction. Many studies follow women for a longer period, as postpartum hypertension and renal impairment may not be fully apparent at six weeks. Since some cases of hypertension resolve spontaneously while others persist and indicate long-term cardiovascular risk, discussing the potential need for extended follow-up would enhance the interpretation of the findings. Another important aspect to clarify is whether the study differentiates between persistent hypertension and transient postpartum hypertension. Blood pressure fluctuations are common in the postpartum period, and it is unclear whether hypertension detected at six weeks was classified as chronic or if resolution was observed in some women. Differentiating between transient and persistent hypertension is crucial, as the long-term cardiovascular implications differ. Addressing this in the discussion would provide a clearer understanding of the clinical significance of the findings.

Response

Thank you. We deliberately choose six weeks postpartum as the endpoint for this study because it is at this timepoint we discharge the postpartum women from the clinic. This timepoint was important for us so that we have a better understanding whether it was safe to continue this practice or change practice. This study makes a case for longer follow-up for some of these women with high cardiovascular risk at this timepoint. The participants are confirmed cases of hypertension which extend into the postpartum period. Being unable to say if hypertension at six weeks postpartum is transient has been included as a limitation. Page 20, paragraph 1.

19. The accuracy of blood pressure measurement methods should also be acknowledged. The study relies on office blood pressure readings, but 24-hour ambulatory blood pressure monitoring is considered the gold standard for detecting true hypertension, particularly in distinguishing between white-coat hypertension and masked hypertension. If ambulatory monitoring was not used, discussing the potential for misclassification would add transparency and highlight a possible limitation. **Response**

Thank you. The limitation of the method of blood pressure measurement used has been noted in the discussion. Page 20, paragraph 1. We do not have facility of 24- hour ambulatory blood pressure measurement.

20. The discussion should include a structured acknowledgment of all the key study limitations. The absence of baseline cardiovascular and renal function is an important limitation, but not the only one. I suggest the authors to consider addressing other important limitations such as the small sample size and its impact on statistical power.

Response

Thank you. All these limitations have been added to the discussion. Page 19, last paragraph and Page 20, first paragraph.

21. Language - I would also suggest a double check for English style and language.

Response

Thank you for your suggestion. We have proofread the final version and corrected grammatical and typographical errors where necessary.

In addition, endnote has been used for reference to minimize errors in reference organization.

VERSION 2 - REVIEW

**Reviewer**	**2**
**Name**	**Kitt, Jamie**
**Affiliation**	**University of Oxford, Department of Cardiovascular Medicine**
**Date**	**23-May-2025**
**COI**	

Recommendation:

Happy to accept with minor corrections as this is an important piece of work in Nigeria that highlights the huge gaps in diagnosis and management in this country/region.

Minor corrections:

Abstract - typo in participants sections: should read '…and blood pressure were collected on admission.'

Grammar - strengths and limitations section:

'None of the participants were (not was) lost to follow up…'

Additional limitation - small number in the early onset PET group limits the interpretation of the findings

Intro

Suggest adding 'or persistent proteinuria' to sentence 'While the risk of renal dysfunction may manifest as deranged serum creatinine…'

Results

Below table 4 - sentence on impact of age and bmi as confounders can be deleted and moved to discussion where it already mentioned anyway

Discussion

Grammar 'similar to what has been reported' used twice on page 19- suggest rewording eg ....as has been previously reported

Conclusion – additional detail needed:

Needs to highlight that over 80% of women were still either classed in prehypertension or hypertension at 6 weeks and that given their is good observational and cohort data , which suggests BP at 6 weeks this links to long term risk of CV disease and adverse CV remodelling, it is vital care in Nigeria focuses on managing blood pressure in this period

VERSION 2 - AUTHOR RESPONSE

Reviewer 2

1. Abstract - typo in participants sections: should read '…and blood pressure were

collected on admission.'

Response

Thank you for drawing our attention to this. The typographical error has been corrected. Page 1, last paragraph.

2. Grammar - strengths and limitations section:
   'None of the participants were (not was) lost to follow up...'
   Response
   Thank you for highlighting this grammatical error. It has been corrected. Page 2, last paragraph.

3. Additional limitation - small number in the early onset PET group limits the interpretation of the findings.
   Response
   Thank you. The limitation has been expanded to capture this statement as follows: In addition, the small number of participants in the early onset preeclampsia group limits the interpretation of the findings. Page 3, Paragraph 1.

4. Intro
   Suggest adding 'or persistent proteinuria' to sentence 'While the risk of renal dysfunction may manifest as deranged serum creatinine...'
   Response
   Thank you. This phrase has been added to the comment as advised. It now reads: While the risk of renal dysfunction may manifest as deranged serum creatinine or persistent proteinuria. Page 4. Paragraph 1.

5. Results
   Below table 4 - sentence on impact of age and bmi as confounders can be deleted and moved to discussion where it already mentioned anyway.
   Response
   Thank you. Affected phrase has been deleted under Table 4. Page 17.

6. Discussion
   Grammar 'similar to what has been reported' used twice on page 19- suggest rewording egas has been previously reported.
   Response
   Thank you. Done. Page 20, Paragraph 2

7. Conclusion – additional detail needed:
   Needs to highlight that over 80% of women were still either classed in prehypertension or hypertension at 6 weeks and that given their is good observational and cohort data , which suggests BP at 6 weeks this links to long term risk of CV disease and adverse CV remodelling, it is vital care in Nigeria focuses on managing blood pressure in this period.

Response

The conclusion has been restructured to capture this suggestion. Page 22, last paragraph. Thank you.
